# Visual Quantitative Characterization of External Corrosion in 3LPE Coated Pipes Based on Microwave Near-Field Reflectometry and Phase Unwrapping

**DOI:** 10.3390/s25165126

**Published:** 2025-08-18

**Authors:** Wenjia Li

**Affiliations:** State Key Laboratory for Strength and Vibration of Mechanical Structures, Shaanxi Engineering Research Center of NDT and Structural Integrity Evaluation, School of Aerospace Engineering, Xi’an Jiaotong University, Xi’an 710049, China; wenjiadyn@stu.xjtu.edu.cn

**Keywords:** three-layer polyethylene coated steel pipeline, phase unwrapping, background subtraction, pipeline corrosion, microwave near-field NDT

## Abstract

Three-layer polyethylene (3LPE) coated steel pipelines are currently the preferred solution for global oil and gas transmission. However, external corrosion beneath the 3LPE coating poses a serious threat to pipeline operations. The pressing concern for pipeline safety and integrity involves non-destructive evaluation techniques for the non-invasive and quantitative interrogation of such defects. This study therefore explores linear frequency-sweeping microwave near-field non-destructive testing (NDT) techniques for imaging and evaluating the pitting corrosion beneath 3LPE coating. An improved branch-cut method is proposed for the high-precision phase unwrapping of the microwave phase image sequence, and its superiority over traditional methods in terms of accuracy and robustness is validated. A background subtraction method based on kernel density estimation (KDE) is presented to suppress the lift-off effect on the pipeline geometry. In addition, the principal-component-analysis-wavelet-based principal component extraction and fusion enhance the detection signal-to-noise ratio (SNR) and image contrast, while mitigating the annular artifacts around the corrosion. The experimental results demonstrate the feasibility of the proposed approach for the detection, imaging, and characterization of external corrosion beneath the 3LPE coating of pipelines.

## 1. Introduction

**Figure 1:**
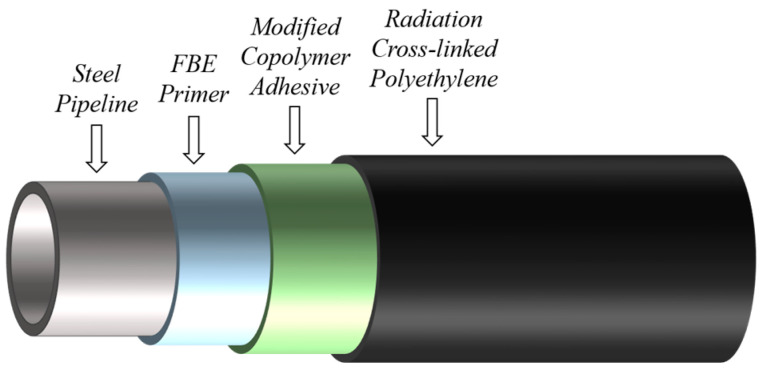
Scheme of the three-layer polyethylene (3LPE) coating structure.

Oil and gas pipelines play an essential role in energy transmission and distribution due to their high reliability and efficiency. As an advanced anti-corrosion technology, the three-layer polyethylene (3LPE) coating system is widely used for pipeline external corrosion resistance, joint patching, and restoration [1,2]. 3LPE is a three-layer functional coating structure, as illustrated in Figure 1: the inner layer consists of a high-performance fusion-bonded epoxy (FBE) primer that fuses with the sand-blasted surface to offer excellent resistance to cathodic delamination; the middle layer consists of a modified copolymer adhesive; and the outer layer of radiation cross-linked polyethylene (RCPE) is used for protection against any physical impact [3]. However, following long-term exposure to harsh service environments, corrosion may occur beneath the 3LPE coating, which is difficult to detect through visual inspection and inevitably damages the pipeline’s structural integrity [4,5].

Over the years, as a recognized effective strategy and solution, non-destructive testing (NDT) has been extensively applied in pipeline corrosion monitoring, identification, and quantification [6]. Magnetic flux leakage testing (MFL) uses a strong magnetic field on the pipeline and, with surface defects causing magnetic flux deflection and overflow, the variations can be monitored using magnetic induction probes [7,8]. Ultrasonic testing (UT) employs ultrasonic generators to guide ultrasonic waves to inspect the specimen, with reflected or scattered energy from defects analyzed by the receiver [9]. Emerging UT technologies, such as electromagnetic acoustic transducers (EMATs) and phased array ultrasonic testing (PAUT), have significantly improved pipeline defect detection capabilities [10,11,12]. Eddy current testing (ECT) relies on electromagnetic induction, where alternating magnetic fields induce currents in conductive samples, and the defect information is inferred by analyzing impedance variations [13,14,15]. Radiographic testing (RT) utilizes radiation sources to penetrate materials, with digital detectors capturing the variations in transmitted radiation to generate images, which facilitate internal defect detection, especially in identifying volumetric defects (e.g., porosity and inclusions) and evaluating the weld integrity in pipelines [16,17,18]. While traditional NDT has conducted in-depth research in advanced signal processing, real-time visualization, novel probe design, and automatic and intelligent system development [19,20], most studies have concentrated on internal corrosion or external defects under thin insulation, which are quite limited in addressing external defects beneath thick protective coatings.

As a cutting-edge NDT technology, microwave testing offers unique advantages of non-contact, high sensitivity, and strong penetration, making it ideal for detection of internal voids and delamination in dielectric materials, as well as corrosion and cracks on metal surfaces [21,22]. Currently, pipeline defect detection via microwave typically uses a cylindrical scanning method, where the probe stays fixed while the pipeline is rotated on a scanning stage. The lift-off distance is maintained to minimize the effect of the pipeline’s geometry during scanning [23,24]. However, this approach is unsuitable for on-site inspections. In addition, while phase information has been proven to be more beneficial than amplitude in detecting tiny defects due to its higher sensitivity [25], it is more vulnerable to detection noise. Additionally, frequent phase wrapping introduces artifacts, leading to misjudgment and greatly hindering its application in defect identification.

A frequency-sweeping microwave near-field NDT method based on phase unwrapping (PU) is thus proposed for evaluating and screening corrosion beneath the 3LPE coating of a pipeline via plane grating scanning. The innovations include the following: (1) an improved branch-cut algorithm based on a self-adaptive genetic algorithm (AdaptGA) is developed and validated for PU of the raw phase image sequence; (2) a background subtraction method based on kernel density estimation (KDE) is provided to mitigate the lift-off effect of the pipeline surface; and (3) principal component extraction and fusion, based on principal component analysis (PCA) and wavelet transform, are applied to enhance detection SNR and evaluation accuracy.

This study explores the feasibility of the proposed method for evaluating external corrosion of pipelines beneath 3LPE coating. The rest of this paper is organized as follows: Section 2 introduces the theoretical background of microwave near-field NDT, followed by a description of the proposed PU algorithm, which is compared and validated against traditional methods in terms of accuracy and noise robustness by numerical simulations in Section 3; Section 4 details the experimental studies, including background subtraction, principal component extraction and fusion using PCA with wavelet transform, and corrosion imaging; Section 5 presents the quantitative evaluation.

## 2. Theory and Methodology

### 2.1. Theoretical Background

**Figure 2:**
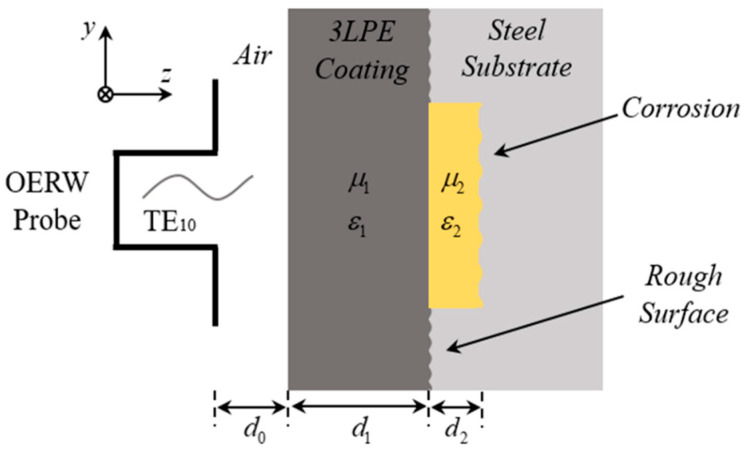
Scheme of microwave interrogation on a metallic substrate with 3LPE coating.

The principle of microwave near-field NDT for corrosion beneath 3LPE coating relies on analyzing the characteristic changes in EM waves reflected or scattered at the interface between the coating and the pipe. As the operating wavelength and waveguide probe size are much smaller than the pipeline radius, this can be approximately equivalent to a stratified dielectric structure backed by a metallic substrate, as shown in Figure 2. For the dominant TE_10_ mode, the excitation aperture field distribution is given by [26]:

(1) $$E_{y}\left(x,y,0\right)={\bar{e}}_{0}\left(x,y\right)=\left\{\begin{array}{l} \sqrt{\frac{2}{ab}}\cos\left(\frac{\pi x}{a}\right),\;\left(x,y\right)\in aperture\\ 0\;\;\;\;\;\;\left(x,y\right)\notin aperture \end{array}\right.$$

where *a* and *b* represent the broad and narrow transverse dimensions of the waveguide. Using transverse vector mode functions and their orthogonal properties, the terminating admittance of the waveguide *Y_WG_* can be constructed using the following equations [27]:

(2) $$Y_{WG}=G+jB=\frac{\int_{aperture}\left[\bar{E}\left(x,y,0\right)\times \bar{W}\left(x,y\right)\right]\cdot {\hat{a}}_{z}dxdy}{{\left[\iint_{aperture}\bar{E}\left(x,y,0\right)\times {\bar{e}}_{0}\left(x,y\right)dxdy\right]}^{2}}$$

(3) $$\bar{W}\left(x,y\right)=\bar{H}\left(x,y,0\right)+\sum_{n=0}^{\infty }Y_{n}{\bar{h}}_{n}\left(x,y\right)\iint_{aperture}\bar{E}\left(\eta ,\xi ,0\right)\cdot {\bar{e}}_{n}\left(\eta ,\xi \right)d\eta d\xi$$

where $\bar{E}$(*x*,*y*,0) and $\bar{H}$(*x*,*y*,0) are the apertures of electric and magnetic field distributions. The nth vector mode functions are *e_n_* and *h_n_*, and *Y_n_* is the characteristic admittance of the waveguide for the nth mode. The interaction between the layered structure and incident waves is rather complex. In general, the complex reflection coefficient Г is a function of the waveguide dimensions, terminating admittance, and electromagnetic parameters of the sample. The Г in the TE_10_ mode at the waveguide aperture is given as follows [28]:

(4) $$\Gamma =\left|\Gamma \right|e^{j\varphi }=\frac{Z_{in}-Z_{WG}}{Z_{in}+Z_{WG}}$$

(5) $$Z_{in}=\sqrt{\frac{j\omega \mu }{\sigma +j\omega \varepsilon_{r}}}$$

where *Z_WG_* represents the waveguide termination impedance (*Z_WG_* = 1/*Y_WG_*), and *Z_in_*, *μ*, *σ*, and *ε_r_* represent the intrinsic impedance, permeability, conductivity, and permittivity of the sample, respectively. |Г| and *φ* are the amplitude and phase. The above equations indicate that the appearance of defects disturbs the surface current distribution of the specimen, thus resulting in equivalent impedance changes near the defects, and higher-order TM modes may be produced due to the interrogation between the waveguide and the sample, all of which will change the microwave reflection and scattering characteristics. By acquiring and analyzing the changes in reflection coefficient, such as amplitude, phase, and time delay, the corrosion information can be deduced in real time.

### 2.2. Proposition of Phase Unwrapping Algorithm

Many applications that rely on phase retrieval, such as interferometric synthetic aperture radar (InSAR) and magnetic resonance imaging (MRI), involve solving the unwrapping problem. The related methods are typically classified into three categories: (1) minimum-norm, (2) path-dependent, and (3) machine learning. Minimum-norm methods, such as the transport of intensity equation (TIE) [29], phase unwrapping max-flow (PUMA) [30], and least squares (LS) [31], estimate the PU via global optimization by minimizing an *L*_p_-norm. By contrast, the general idea of path-dependent methods is to perform the line integration along a certain path based on residual search. The classic methods include Goldstein’s branch cut algorithm [32], sorting by reliability following a non-continuous path (SRNCP) [33], and quality-guided (QGPU) [34], both of which adopt different path selection strategies. Goldstein’s method offers the benefits of fast PU speed and high accuracy under a high SNR; however, the main drawbacks are as follows: (1) the branch cuts are prone to overcross and form “isolated islands” in dense residue regions, and (2) the cuts fail to effectively block the propagation of local errors under high-noise conditions. An improved branch cuts method based on AdaptGA is thus proposed:

#### 2.2.1. Residue Identification

Goldstein’s method describes the phase unwrapping as a line integral process of the wrapped-phase gradient, as follows [35]:

(6) $$\Psi \left(r\right)=\int_{C}\nabla \Phi \left(r\right)\cdot dr+\Psi \left(r_{0}\right)$$

where Ψ denotes the unwrapped phase, Φ denotes the wrapped-phase gradient, and *C* denotes any integral path between **r_0_** and **r**. The residues are identified by a four-point loop integral; i.e., a pixel (*i*, *j*) on the wrapped-phase Φ is a residue if the sum *R*(*i*, *j*) of the gradient around each 2 × 2-pixel closed loop is nonzero. The formula is as follows:

(7) $$R\left(i,j\right)=W\left[\Phi_{i+1,j}-\Phi_{i,j}\right]+W\left[\Phi_{i+1,j+1}-\Phi_{i+1,j}\right]+W\left[\Phi_{i,j+1}-\Phi_{i+1,j+1}\right]+W\left[\Phi_{i,j}-\Phi_{i,j+1}\right]$$

where *W* (·) represents the wrap factor. Residue polarity depends on *R*(*i*, *j*) (positive or negative), and the corresponding residue distribution map is subsequently generated.

#### 2.2.2. Residue Pre-Processing

An improved nearest-neighbor strategy is introduced to pre-process the residues based on their distribution characteristics in the residue map, which significantly reduces the time and space complexity of subsequent branch-cut optimization using AdaptGA. The specific steps are as follows:

- Step 1. Take every residue as the center, search for the opposite residue from left to right and top to bottom in a 3 × 3-pixel diamond search box, place the cut as balanced and marked, and search for the next one until all the residues are processed.
- Step 2. If the box contains an image boundary, connect to it; if there is no opposite polarity residue in the search box, skip to the next residue and return to Step 1.
- Step 3. Check whether the rest positive and negative residues are equal (*N_pos_* = *N_neg_*). If so, turn to Step 5. If there are not equal, proceed to Step 4.
- Step 4. Monopole compensation. If *N_pos_* > *N_neg_*, calculate the closest distance of each positive residue to the image boundaries and sort them in ascending order; balance the first *N_pos_*–*N_neg_* with the nearest boundary, and vice versa; then turn to Step 5.
- Step 5. Check whether the number of the remaining dipoles is smaller than the optimization threshold *N_opt_*. If so, end the pre-processing; if not, increase the box size by 2 pixels, and return to Step 1 for a new search iteration.

After pre-processing the residues, the unwrapping problem is transformed into finding the global optimum by minimizing the total branch-cut length for the remaining residues based on minimum-cost-matching (MCM) graph theory [36], which is analogous to the classical traveling salesman problem (TSP) [37].

#### 2.2.3. Population Initialization and Selection Operation

The optimization should be first coded in GA syntax form. Different chromosomes represent different negative residue sequences and cut lengths. Fix the positive residue sequences and optimize the negatives. Set the population size to 1000 and the maximum generation to 100. Calculate the Euclidean distance between each residue pair, and sum them as shown in Equation (8). The fitness function is the reciprocal of the total length:

(8) $$Length=\sum_{i=1}^{N}\sqrt{{\left(x_{i}^{+}-x_{i}^{-}\right)}^{2}+{\left(y_{i}^{+}-y_{i}^{-}\right)}^{2}}$$

where (*x_i_*^+^, *y_i_*^+^) and (*x_i_^−^*, *y_i_*^−^) are the *i-th* coordinates of positive and negative residue. The selection operation employs an elitism strategy. Sort chromosomes by fitness, remove low-fitness ones, add high-fitness ones in equal numbers, and shuffle the sequence. Use stochastic universal sampling to prevent local optima and over-selection.

#### 2.2.4. Adaptive Crossover and Mutation Operation

**Figure 3:**
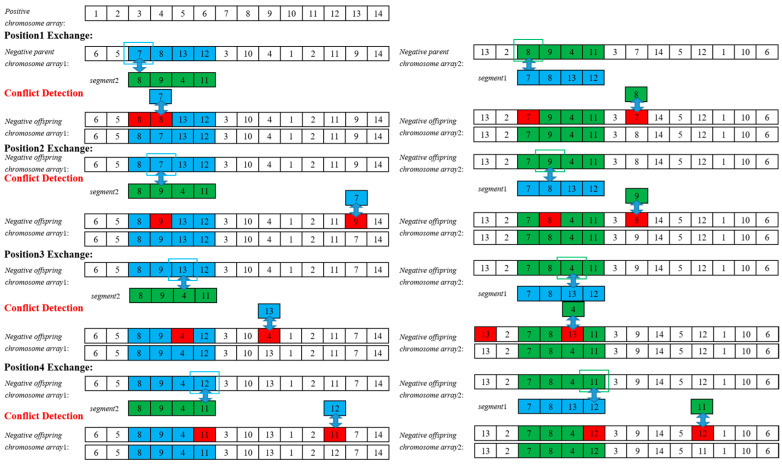
Scheme of adaptive crossover operation. Parent 1’s gene segments for adaptive crossover are shown in blue, parent 2’s segments in green, and the genes to be exchanged are marked in red.

Adaptive crossover operation. To ensure high-quality chromosomes and prevent identical genes in the offspring, the neuron sigmoid activation function and partially matched crossover are applied, as illustrated in Figure 3. The steps include the following:

- Step 1. Select two chromosomes sequentially, calculate their fitness values, and compute the crossover rate *P_c_* using the larger fitness *f_c_*, as shown in the formula below:

(9) $$P_{c}=\left\{\begin{array}{cc} \frac{P_{c}^{\max}-P_{c}^{\min}}{1+\exp\left(-c_{0}+\frac{2c_{0}\left(f_{c}-f_{avg}\right)}{f_{\max}-f_{avg}}\right)}+P_{c}^{\min} & ,f_{c}\geq f_{avg}\\ P_{c}^{\max} & ,f_{c}<f_{avg} \end{array}\right.$$

where *P_c_^max^* = 0.9 and *P_c_^min^* = 0.6 denote the upper and lower bounds of the adaptive crossover rate, and *f_max_* and *f_avg_* denote the maximum and average fitness, respectively.

- Step 2. Generate a random number. If it is less than *P_c_*, perform the crossover operation; otherwise, copy the two parents to the offspring and return to Step 1.
- Step 3. Randomly select a crossover region, and exchange the corresponding genes.
- Step 4. Conflict detection. If the genes in the crossover region conflict with others, replace them with genes from the original positions to produce the new offspring.

**Figure 4:**
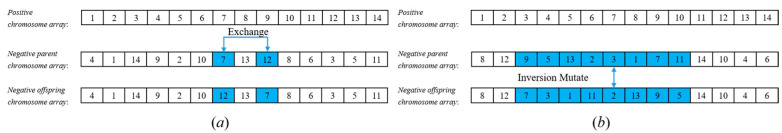
Schemes of (**a**) adaptive exchange mutation, and (**b**) adaptive inversion mutation.

Adaptive exchange mutation operation. The linear probability activation function is chosen due to the low exchange mutation rate, as shown in Figure 4a. The steps include the following:

- Step 1. Select a chromosome sequentially from the population and calculate its fitness value *f_em_*. Then, compute the exchange mutation rate *P_em_* using Equation (10):

(10) $$P_{em}=\left\{\begin{array}{cc} P_{em}^{\max}-\frac{\left(P_{em}^{\max}-P_{em}^{\min}\right)\left(f_{em}-f_{avg}\right)}{f_{\max}-f_{avg}} & ,f_{em}\geq f_{avg}\\ P_{em}^{\max} & ,f_{em}<f_{avg} \end{array}\right.$$

where *P_em_^max^* = 0.005 and *P_em_^min^* = 0.001 are the range limits of the exchange mutation rate.

- Step 2. Generate a random number. If it is less than *P_em_*, perform the exchange mutation; otherwise, copy the parent genes to the new offspring and return to Step 1.
- Step 3. Randomly select two positions, and exchange genes to obtain the new offspring.

Adaptive inversion mutation operation. The cosine probability activation function is chosen to perform the inversion mutation, as shown in Figure 4b. The steps include the following:

- Step 1. Select a chromosome sequentially from the population and calculate the fitness value *f_im_*, then calculate the inversion mutation rate *P_im_* using Equation (11):

(11) $$P_{im}=\left\{\begin{array}{cc} \frac{P_{im}^{\max}+P_{im}^{\min}}{2}+\frac{P_{im}^{\max}-P_{im}^{\min}}{2}\cos\left(\frac{f_{im}-f_{avg}}{f_{\max}-f_{avg}}\right) & ,f_{im}\geq f_{avg}\\ P_{im}^{\max} & ,f_{im}<f_{avg} \end{array}\right.$$

where *P_im_^max^* = 0.08 and *P_im_^min^* = 0.05 denote the range limits of the inversion mutation rate.

- Step 2. Generate a random number. If it is less than *P_im_*, perform an inversion mutation; otherwise, copy the parent genes to the new offspring, and return to Step 1.
- Step 3. Randomly select a mutation region, and sort the genes to obtain offspring.

#### 2.2.5. Update Population and Phase Integral

**Figure 5:**
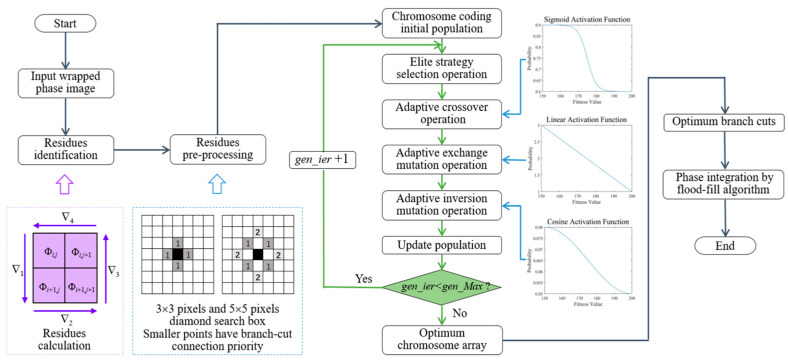
The flowchart of the improved branch-cut algorithm based on AdaptGA.

Chromosomes with low fitness are replaced by those with higher fitness from the old population to generate a new population. By repeatedly applying elitism-strategy selection, adaptive crossover, and mutation operations, the optimal branch cuts are gradually achieved. The phase is unwrapped by computing the integral of the wrapped-phase gradient pixel by pixel using flood-fill algorithm. The overall flowchart of the proposed unwrapping algorithm is illustrated in Figure 5.

## 3. Numerical Simulations of the Phase Unwrapping Algorithm

**Figure 6:**
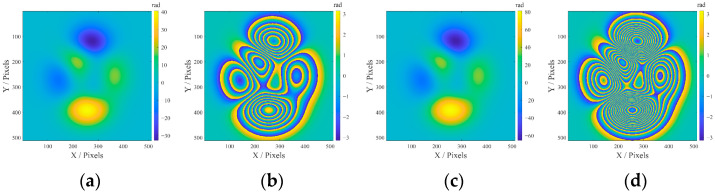
Ideal simulated phase images in radians: (**a**,**b**) denote 5-peak unwrapped and wrapped images, and (**c**,**d**) denote 10-peak unwrapped and wrapped images, respectively.

The performance of the proposed algorithm is evaluated by two ideal 512 × 512-pixel phase images (both unwrapped and wrapped) with two different Peak-to-Valley values generated by the Matlab^®^ Peaks function to simulate different phase gradients, as shown in Figure 6. Three levels of White Gaussian Noise (WGN) with mean *μ*_noise_ = 0 and standard deviation *σ*_noise_ = 0.3, 0.5, and 0.8 are added to assess the noise robustness. Comparisons are conducted with three classical methods: TIE, SRNCP, and CPULSI [38].

**Figure 7:**
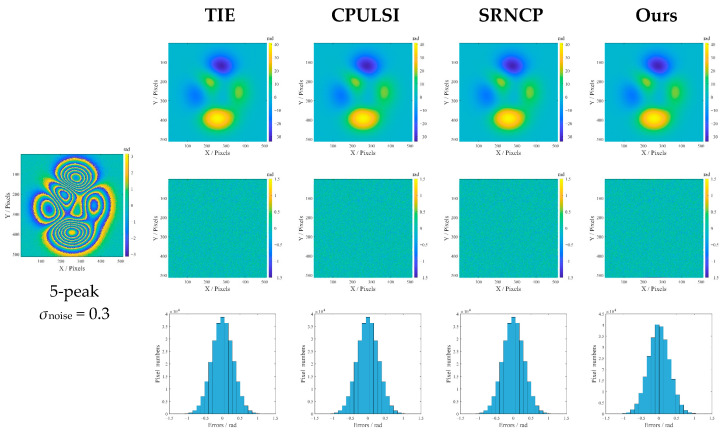
Unwrapped results (5-peak, *σ*_noise_ = 0.3) in radians: the second row to the last row refer to the unwrapped-phase images, the corresponding error maps and the error histograms.

Initially, a series of comparative investigations is conducted under mild noise and low gradient (5-peak, *σ*_noise_ = 0.3), and the resultant unwrapped images, error distribution maps, and error histograms are shown in Figure 7. It is apparent that, although all the methods have achieved good results, there are still slight differences in details. The contour edges of TIE are relatively blurred, while CPULSI and SRNCP show better edge retention. Ours is closest to the ideal without notable differences. The error map reveals that TIE has the lightest spots, while ours shows a more uniform distribution. As for the histograms, although all distributions are approximately Gaussian, TIE shows a wider range with a lower peak, and CPULSI and SRNCP are superior to TIE in error concentration. Ours exhibits the narrowest distribution and highest peak, indicating the lowest error deviation.

**Figure 8:**
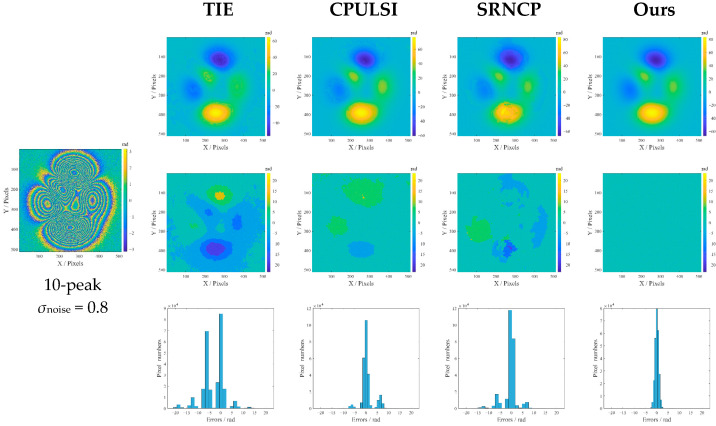
Unwrapped results (10-peak, *σ*_noise_ = 0.8) in radians: the second row to the last row refer to the unwrapped-phase images, the corresponding error maps and the error histograms.

Further comparative studies are implemented under the simulated high noise and phase gradient (10-peak, *σ*_noise_ = 0.8), and the results are shown in Figure 8. It can be seen from Figure 8 that TIE exhibits obvious phase distortion with the largest dark areas in the error map, especially around high-gradient regions; its multi-peak histogram with the widest range indicates that most pixels have large unwrapping errors. Large noticeable bright areas also appear in the error map of CPULSI and, although its histogram peak is relatively higher, a certain number of errors still distribute over a medium-width interval. SRNCP reveals phase discontinuities and local contour anomalies, with a more dispersed color distribution in the error map and multi-peak histogram. In contrast, our method achieves the most accurate phase retrieval, particularly in high-gradient regions, as demonstrated by a uniform error map that reveals minimal unwrapping errors, as well as the highest peak histogram. This indicates excellent noise immunity and high precision in handling complex phase wrapping problems under high noise interference.

To further quantitatively evaluate the unwrapping accuracy, two metrics are subsequently employed for comparison, namely, image root mean square error (RMSE) and signal-to-noise ratio (SNR), which are formulated as Equations (12) and (13), respectively.

(12) $$RMSE=\sqrt{\frac{1}{M\times N}\sum_{i=1}^{M}\sum_{i=1}^{N}{\left[\psi \left(i,j\right)-\hat{\psi }\left(i,j\right)\right]}^{2}}$$

(13) $$SNR=10\log_{10}\left[\frac{\sum_{i=1}^{M}\sum_{i=1}^{N}{\left[\psi \left(i,j\right)\right]}^{2}}{\sum_{i=1}^{M}\sum_{i=1}^{N}{\left[\psi \left(i,j\right)-\hat{\psi }\left(i,j\right)\right]}^{2}}\right]$$

where $\hat{\psi }\left(i,j\right)$ and *ψ*(*i*, *j*) represent the unwrapped and ideal phase at pixel(*i*, *j*), while *M* and *N* are the number of rows and columns of the unwrapped-phase images, respectively. The quantitative comparison in Table 1 shows that both noise intensity and phase gradient jointly determine the unwrapping result. The RMSE and SNR are nearly identical under low-noise conditions, which suggests that all methods can stably unwrap the phase with no significant differences. However, the performance among the four methods is significantly different in scenarios with medium to high noise and complex phase structures. Our method offers a higher SNR and accuracy, with up to 49% RMSE reduction and 18% SNR improvement (10-peak, *σ*_noise_ = 0.8), showing superior PU reliability.

Regarding the computation cost, all the methods are implemented in Figure 8 using a PC with Intel Core i7-7700HQ CPU @2.80 GHz and 32 GB RAM (Intel, Santa Clara, CA, USA). The average execution time is 0.261 (TIE), 0.449 (CPULSI), 0.867 (SRNCP), and 3.283 (Ours) seconds. While the proposed PU algorithm demonstrates better accuracy and robustness, it is important to acknowledge that the conservative iterative strategy used to ensure precision results in higher costs during the stage of feasibility confirmation. The balance between accuracy, robustness, and computational efficiency is crucial for subsequent optimization.

## 4. Experiment and Discussion

### 4.1. Experimental System Setup

**Figure 9:**
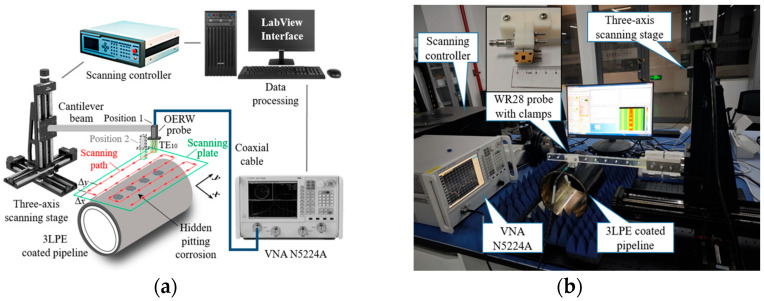
Microwave near-field NDT system: (**a**) schematic illustration and (**b**) photograph.

Experiments were conducted to investigate the imaging and evaluation of the hidden corrosion using microwave reflectometry. The established near-field microwave NDT system includes a Keysight N5224A vector network analyzer (Keysight, Santa Rosa, CA, USA), a WR28 waveguide probe with clamps, a three-axis mechanical scanning stage, and a PC with a scanner controller using LabVIEW. The probe is secured to the cantilever arm via custom-designed clamps to ensure positional stability during translation. The schematic illustration and a photograph of the system are portrayed in Figure 9. The probe is mounted on a mechanical stage and performed 2-D grating frequency-sweeping inspection, the scanning interval is fixed at 1 mm, and the number of frequency samples is 21, with a standoff distance of 1 mm to ensure enough inspection sensitivity. The scanning procedure is performed sequentially in a controlled laboratory environment: at each predefined spatial position, the frequency is swept across the designated band. The reflection coefficient (S11 or Г) varies with lift-off distance and metal discontinuities beneath the coating, and these variations are captured in real time by VNA before moving the probe to the next position.

### 4.2. Specimen Preparation

**Figure 10:**
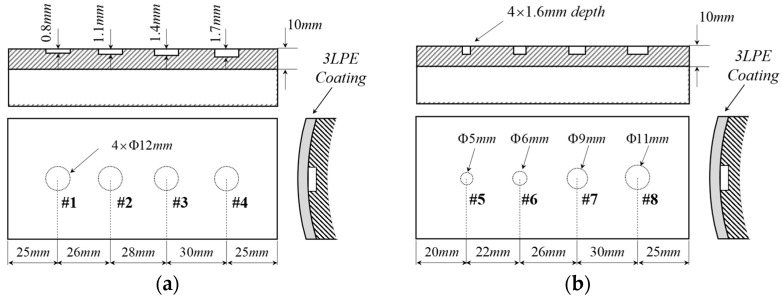
Specimens with artificial flat-bottom holes: (**a**) specimen I and (**b**) specimen II.

This preliminary feasibility study utilizes controllable and quantifiable artificial machined defects (flat-bottom holes, FBHs) to simulate pitting corrosion. FBHs are fabricated along the axis direction on the outer surface of two seamless carbon steel pipes DN200 with Φ 219 × 10 mm, which are typical carbon structural pipes in the petroleum and gas industry. Specimen I has FBHs with depths of 1.6 mm and diameters ranging from 3 mm to 9 mm, while specimen II has a fixed diameter of 12 mm and depths varying from 0.8 mm to 1.7 mm. The geometric parameters are shown in Figure 10. Steel pipelines are first preheated to 60–80 °C after external surface pre-treatment for cleaning and roughness, and then the FBE primer is applied with a thickness of 0.4–0.6 mm. Standard RCPE tape with 1.2 mm PE thickness and 1.0 mm copolymer adhesive is tightly wrapped around the pipeline after the adhesive is melted, ensuring the outer surface is free of wrinkles and bulges. After the coating has cooled, the peel test and impact check are conducted to meet the GB/T 23257-2017 [39] standards for corrosion resistance grade.

### 4.3. Frequency Band Selection

To determine the appropriate operating frequency range in microwave Ka-band, several interconnected factors should be taken into account: (1) Coating properties. The thickness and electromagnetic properties (such as permittivity, loss tangent, etc.) of 3LPE coating directly influence the propagation, attenuation, and reflection/transmission characteristics of the microwave, as described in Section 2.1; (2) Spatial resolution. Higher frequencies correspond to shorter wavelengths, offering finer spatial resolution and improved accuracy, crucial for identifying small defects; and (3) Penetration depth. In microwave engineering, the attenuation of microwave in low lossy dielectric materials (tan *δ* ≪ 1) mainly comes from dielectric loss, and the skin depth can be given by the following [40]:

(14) $$\delta \approx \frac{c}{\pi f\sqrt{\varepsilon_{r}}\tan\delta }$$

where tan *δ* represents the loss tangent of dielectric coating. It is clear from Equation (14) that, although the low-frequency range of the Ka-band offers better coating penetration and more significant signal response, spatial resolution is compromised. However, as the frequency increases, microwaves undergo faster energy attenuation within the polymer, weakening the reflected signal’s amplitude and inevitably reducing detection accuracy. Therefore, this study selects 36.0–38.0 GHz as the appropriate operating band, based on the balance between theoretical analysis and practical detection performance.

### 4.4. Experimental Validation of the Phase Unwrapping Algorithm

**Figure 11:**
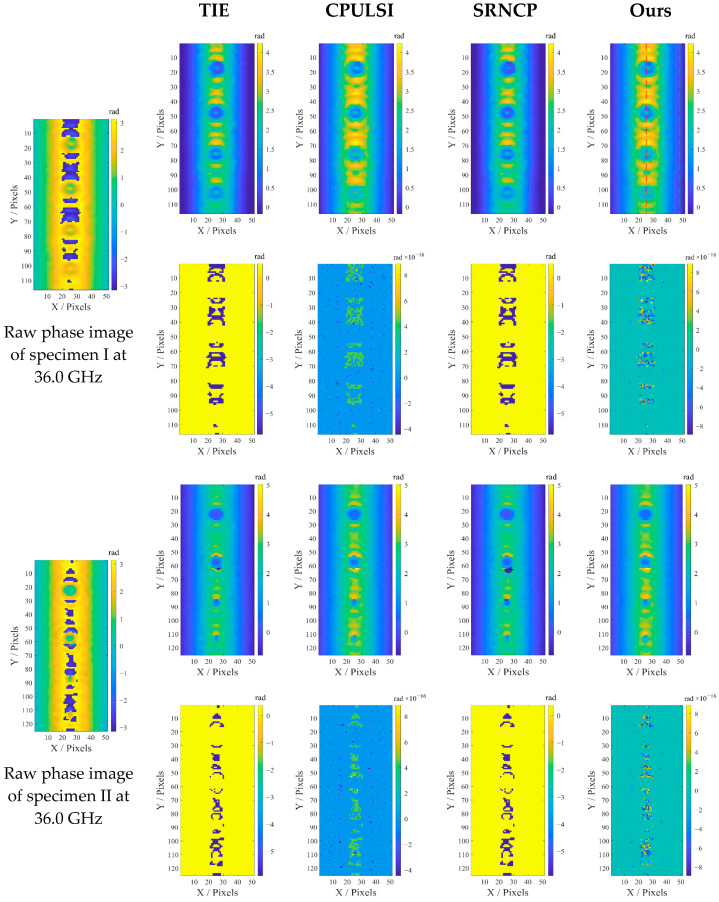
Unwrapped results for two raw phase images: rows 1 and 3 show method results; rows 2 and 4 show error maps. Dashed data are used for background reconstruction in Section 4.5.

The original experimental data is collected after the completion of grating scanning. Considering the trade-off between spatial resolution, sensitivity, and penetration depth mentioned in Section 4.3, the raw phase images at 36.0 GHz are chosen for presentation in the first column of Figure 11. It is evident from the original phase images of Figure 11 that the extensive wrapping of the phase and lift-off noise caused by the geometric features of the pipeline severely hinder the accurate identification of the corrosion profile, particularly in the area of corrosion openings. The unwrapping performance of the four methods in Section 3 is compared. Note that the error distribution map represents the difference between the raw wrapped and rewrapped images after unwrapping. The results reveal that both TIE and SRNCP exhibit significant error regions around the defects, indicating inadequate accuracy at defect edges or in regions with steep phase gradients. While CPULSI provides smoother results, the large green regions in the error map suggest the localized distortion and error concentration. In contrast, our method performs best in phase consistency and detail preservation, with no apparent phase discontinuities or pseudo-gradients, resulting in better suitability for high-precision phase retrievals.

**Figure 12:**
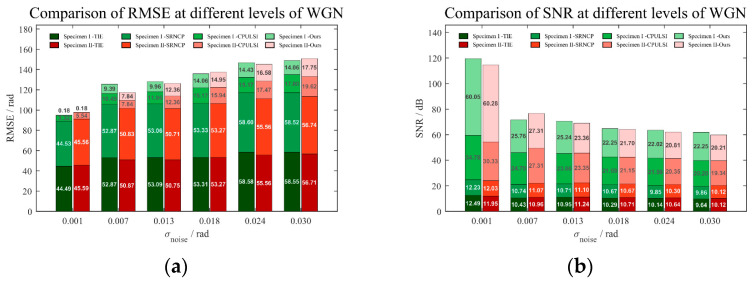
Comparison of metrics under different noise levels and methods: (**a**) RMSE and (**b**) SNR.

To analyze and compare the accuracy and noise resilience of different algorithms, WGN is introduced into the original wrapped experimental image sequence, simulating broadband random interference typical of real-world environments. For noise level selection, an adequate target SNR (SNR > 20 dB) is necessary for detecting pitting corrosion using microwave near-field NDT. Meanwhile, the effect of noise is limited due to strong reflections at the coating-metal interface. WGN is thus applied with *μ_noise_ =* 0, and *σ_noise_* ranges from 0.001 to 0.03 to cover various noise intensities. This range represents challenging yet feasible scenarios, ensuring meaningful comparisons of algorithm testability without overwhelming defect information, particularly in low-relief phase regions [41]. The original phase images (36.0 GHz) of two specimens are unwrapped, and the SNR and RMSE comparison results are presented in Figure 12. In general, all algorithms exhibit an expected increase in RMSE and a corresponding decline in SNR as noise rises. However, our method consistently achieves the lowest RMSE and highest SNR across all noise levels with minimal fluctuation, indicating better stability. Specifically, under low noise conditions (close to the experimental environment), ours achieves an RMSE of 0.18 rad, significantly lower than others, and with an SNR above 60 dB, more than twice that of CPULSI. In contrast, SRNCP and TIE exhibit notably higher RMSE, resulting in greater error accumulation as noise rises. The above analysis demonstrates that our method achieves superior unwrapping accuracy, interference resistance and generalization capability, highlighting its potential for practical applications.

### 4.5. Background Subtraction

In contrast to the cylindrical scanning of pipelines, where the detection signal from any defect-free area remains consistent due to the constant lift-off distance, the lift-off noise may have a significant impact on defect assessment when plane grating scanning is applied in non-Cartesian planes (e.g., cylindrical). We propose a background subtraction method based on kernel density estimation for microwave near-field NDT. This method reconstructs the entire background from the unwrapped image, after which the differential image is directly extracted. This method is founded on the following hypotheses: (1) although the lift-off distance along the x-direction varies during scanning, as shown in Figure 9, it remains unchanged along the axial path; and (2) detection data along the axis mainly originates from a non-defect background due to the sparse distribution of defects. The probability density of the unwrapped phase from each axial path is thus obtained, with the peak of the probability density function corresponding to the background phase. For each phase *φ* to be estimated, the KDE of all known *φ*_i_ at *φ* is calculated via Equation (15):

(15) $${\hat{f}}_{h}\left(\varphi_{i}\right)=\frac{1}{nh}\sum_{i=1}^{n}K\left(\frac{\varphi -\varphi_{i}}{h}\right)$$

where *h* denotes the bandwidth, and *K*(*u*) = 3(1 − *u*^2^)/4 denotes the Epanechnikov kernel function. The log-likelihood function, as shown in Equation (16), is applied to evaluate the KDE fitting of the phase due to its superiority in capturing phase density. The *h* is optimized by leave-one-out logarithmic likelihood cross-validation criterion [42], and the formula is given by Equation (17). By fitting all known phases with KDE, the estimated phase *φ*, corresponding to the curve peak, represents the background of the phase along the axis.

(16) $$Log\text{-}likelihood\left(h\right)=\sum_{i=1}^{n}\log{\hat{f}}_{h}\left(\varphi_{i}\right)$$

(17) $$LLCV\left(h\right)=\frac{1}{n}\sum_{i=1}^{n}\log{\hat{f}}_{h,-i}\left(\varphi_{i}\right)$$

**Figure 13:**
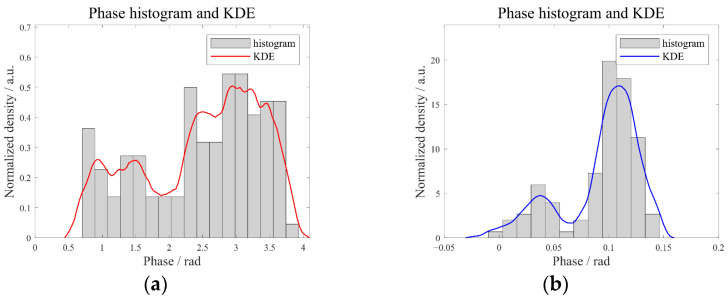
The fitting results of phase histogram by KDE: (**a**) red line and (**b**) blue line.

The unwrapped-phase data from the red and blue lines in Figure 11 (representing defective and non-defective paths) are used to plot the histogram and KDE curve shown in Figure 13. It is clear that the KDE curve provides a good fit to the phase histogram and accurately captures the multi-peak nature. In dense regions, there are no major deviations while, in sparse regions, the curve preserves edge details without over-smoothing.

**Figure 14:**
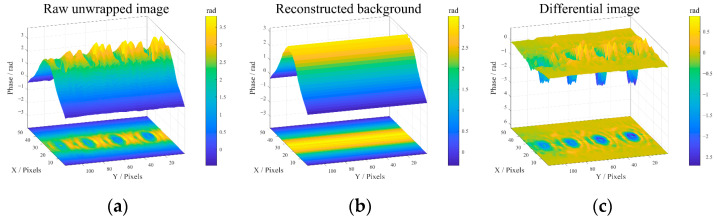
The background subtraction results for specimen I at 36.0 GHz: (**a**) the raw unwrapped- phase image, (**b**) the reconstructed pipeline background, and (**c**) the differential phase image.

The result of background subtraction is shown in Figure 14. Clearly the lift-off effect caused by the pipeline has been effectively suppressed. Our method overcomes the impact of pipeline curvature and highlights defect details by isolating defects from the background in the differential phase image, which provides a solid foundation for the subsequent defect quantitative evaluation in Section 5.

### 4.6. Principal Components Extraction and Fusion

The differential phase image sequence obtained after background subtraction can be regarded as a statistical dataset represented by a tensor. Accurately and efficiently extracting defect information from the sequence with redundant information is crucial. PCA is used to project the multidimensional statistical data into orthogonal subspaces to extract dominant features, and linear transformations are applied to reduce data dimensionality. PCA can be performed via singular value decomposition (SVD) or eigenvalue decomposition (EVD); the discrepancy is that SVD directly decomposes the matrix without needing to compute the covariance matrix, which ensures better numerical stability in processing large-scale datasets.

**Figure 15:**
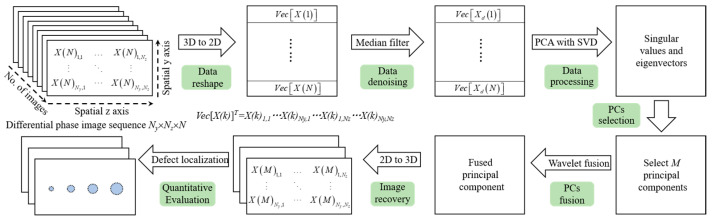
Flow diagram of PCA-wavelet-based principal component extraction and fusion.

The implementation process is detailed in Figure 15. The original differential phase image sequence is vectorized into a reshaped matrix *X* of dimensions *N* × (*N_y_* × *N_z_*), which is then centered and standardized to obtain *X_s_*. SVD factorizes *X_s_* into three matrices *X_s_* = *USV^T^*, the orthogonal matrix *U* captures spatial information in orthogonal spaces, and the diagonal matrix *S* contains singular values of *X_s_*, arranged in descending order of variance to highlight the importance of corresponding principal components (PCs) in matrix *V*, where only the first few principal eigenvectors are selected based on the physical interpretation of microwave interrogation on the 3LPE coated pipeline.

The individual and cumulative contribution rates of the first five components are shown in Table 2. It can be seen that the cumulative contribution of PC1, PC2, and PC3 for specimen I increases from 81.37% to 95.28%, while for specimen II, it increases from 70.84% to 91.00%. As a result, these three components can be taken as the PCs.

**Figure 16:**
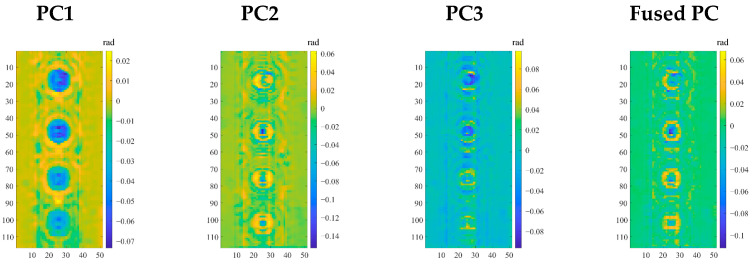
The first three principal components (PCs) and the fused PC in specimen I.

The PC features for specimen I are shown in Figure 16. It is observed from Figure 16 that the four distinct circular defects illustrated by PC1 highlight the presence and position of the corrosion. As the PC with the highest variance in the image sequence, PC1 represents the primary feature of the overall impact of the metal pipeline on the microwave reflection signal. However, it is noted that the reflective disturbances at the defect boundaries generate higher-order TM modes, leading to significant diffraction phenomena around the edges of corrosion. PC2 captures the secondary variance reflection characteristics due to the defect contour during the microwave propagation on the pipeline surface. Unlike PC1, the annular artifacts around the artificial FBHs in PC2 are less noticeable. PC3 exhibits relatively smooth overall color variation, which reflects the edge and detail features of defects as well as the outer surface and interlayer reflection of the 3LPE coating. From a statistical analysis standpoint, the orthogonality of PCs captures the independent defect information, while their complementarity facilitates a more thorough defect characterization. We thus fuse PC2 and PC3 using wavelet transform to better preserve the contours and details of defects, while minimizing the impact of annular artifacts.

After converting the images of PC2 and PC3 to double-precision, a principal component fusion strategy based on 2D discrete wavelet transform (DWT) is proposed. The db4 wavelet is employed as its basis function due to its advantages in time-frequency localization and multi-resolution decomposition stability [43]. The wavelet decomposition is performed at level 2. Notably, low-frequency coefficients are fused using the weighted average (weight of 0.5) to preserve background uniformity and reduce redundancy. In contrast, high-frequency coefficients are fused using the maximum absolute value to highlight the critical texture and edge details. The fused PC obtained by performing the 2D inverse discrete wavelet transform (IDWT) is portrayed in the fourth column of Figure 16. It is clearly observed that the fused image enhances the edge recognition while preserving the original details, and the overall contrast of the defect area is more prominent compared to PC2. As a result, the fused PC with more complete defect information is employed for the following differential phase image reconstruction.

### 4.7. Corrosion Imaging

**Figure 17:**
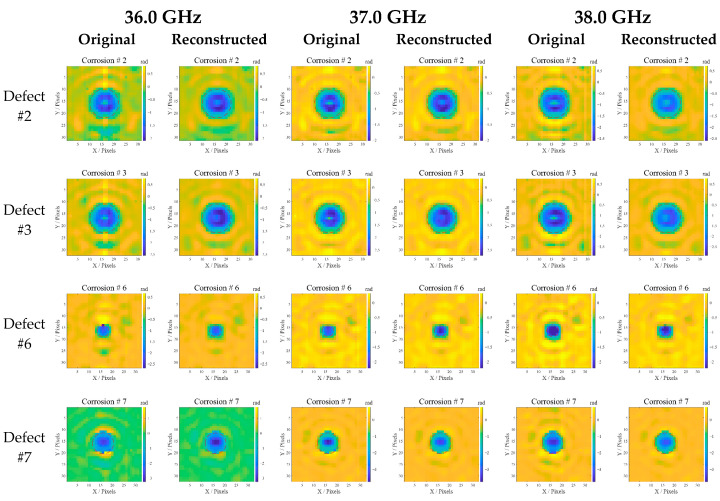
Comparison between original and reconstructed differential phase images. The red dotted circles represent the contour of the ideal images.

The original and reconstructed differential phase images of contrasting corrosion at various frequencies are portrayed in Figure 17. As shown in Figure 17, all images at different detection frequencies distinctly reveal the specific locations and shapes of defects. The reconstructed images exhibit better defect contour integrity compared to the originals, such as defect #3 and #7. Further observation reveals that the image sharpness and details vary at different frequencies. With the frequencies rising from 36 GHz to 38 GHz, the image background becomes more uniform (noise decreases), and defect edges become sharper. High frequencies are ideal for tiny defects, such as defect #6, as it may offer higher resolution; low frequencies are more effective for deep defects due to its stronger penetration ability. The frequency choice can affect the reconstructed image’s accuracy, possibly due to the penetration or scattering properties of microwave interrogation between the waveguide and the corrosion beneath the 3LPE coating. It is noted that almost all defects in the reconstructed images are cleaner than the original, with sharper edges and improved contrast, which confirms the effectiveness of our method in suppressing annular artifacts and removing background noise caused by pipeline roughness.

**Figure 18:**
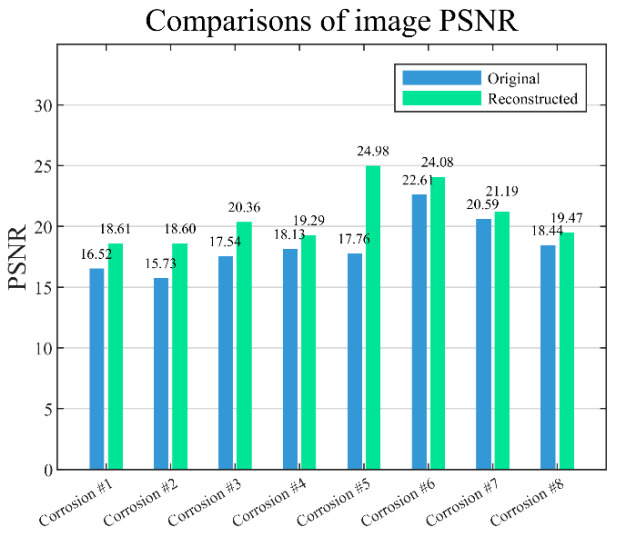
Comparisons of PSNR for the original and reconstructed differential phase images.

To quantitatively assess the image quality reconstructed by principal component fusion, the evaluation metric, peak signal-to-noise ratio (PSNR), is calculated for each reconstructed image at 36.0 GHz using Equation (18). A higher PSNR signifies better reconstruction, effectively preserving the image details and information:

(18) $$PSNR=10\log_{10}[\frac{255^{2}\times M\times N}{\sum_{i=1}^{M}\sum_{j=1}^{N}{\left[g\left(i,j\right)-\hat{g}\left(i,j\right)\right]}^{2}}]$$

where *g* denotes the ideal image, $\hat{g}$ denotes the normalized original or reconstructed differential phase image, and *M* and *N* represent the number of pixels in the image’s rows and columns, respectively. Note that the ideal image is created with pixel values of 1 for defective areas and 0 for defect-free areas, representing the true defect profile. The PSNR comparison is portrayed in Figure 18, showing that all PSNRs of the reconstructed images are higher than the originals, indicating that the reconstruction method effectively suppresses background noise and enhances the image structure. It is noteworthy that the PSNR performance varies with contrasting corrosion. This may be attributed to the defect profile, depth, and the microwave reflection characteristics.

## 5. Defect Quantitative Evaluation

The threshold segmentation of the reconstructed differential phase image is performed using the maximum entropy method [44] to evaluate the amount and distribution of artificial corrosion in the 3LPE coated pipeline. This method utilizes the grayscale distribution of the image to find the optimal threshold that maximizes the total information entropy. The reconstructed image is normalized to [0, 1], scaled to [0, 255] as uint8 data type, and then analyzed to calculate the probability distribution of each grayscale value. For each threshold *t* (*t* = 1, …, 255), the total entropy *H_total_* (*t*) is represented as follows:

(19) $$H_{total}\left(t\right)=H_{f}\left(t\right)+H_{b}\left(t\right)$$

where *H_f_* (*t*) and *H_b_* (*t*) denote the foreground and background entropy, respectively. The optimal threshold t* is determined by maximizing *H_total_* (*t*) using Equations (20) and (21) as follows:

(20) $$t^{*}=\arg\underset{1\leq t\leq L-1}{\max}\left[\log\left(\omega_{f}\cdot \omega_{b}\right)-\frac{E_{f}\left(t\right)}{\omega_{f}\left(t\right)}-\frac{E_{b}\left(t\right)}{\omega_{b}\left(t\right)}\right]$$

where, the *ω_f_*, *ω_b_*, *E_f_*(*t*) and *ω_f_* (*t*) above are given by:

(21) $$\left\{\begin{array}{l} \omega_{f}\left(t\right)=\sum_{i=0}^{t}p_{i}\;\;\;E_{f}\left(t\right)=\sum_{i=0}^{t}p_{i}\log p_{i}\\ \omega_{b}\left(t\right)=\sum_{i=t+1}^{L-1}p_{i}\;\;\;E_{b}\left(t\right)=\sum_{i=t+1}^{L-1}p_{i}\log p_{i} \end{array}\right.$$

**Figure 19:**
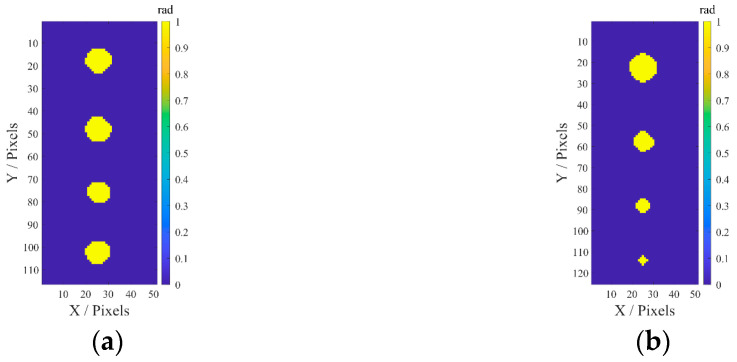
Threshold-segmented binary images of (**a**) specimen I and (**b**) specimen II.

In the equations above, *ω_f_* and *ω_b_* denote the cumulative probabilities of foreground and background, respectively, and *p_i_* denotes the probability of the grayscale value *i* appearing. The threshold segmentation results are shown in Figure 19. It is clear that the size, contours, and distribution of the corrosion depicted in the images closely match the expected design. It is important to note that for specimen 2, as the defect size decreases, the contours of the defect become increasingly indistinct and irregular, particularly for Corrosion #5. This is due to the applicability constraints of waveguide probes in detecting coated pipelines, influenced by factors such as coating properties, detection frequency, and noise. As a result, there are inherent limits in characterization, including the maximum detectable coating thickness, and the minimum detectable defect size and depth.

Following the threshold segmentation, the localization of the artificial corrosion in this study is assessed according to the relative position of the centroid of each defect region. Each centroid coordinate is extracted using the Regionprops function in Matlab^®^ and subsequently compared with the ideal defect positions. The comparative results are tabulated in Table 3, where the maximum relative error of the centroid coordinates is 2.25%, indicating the effectiveness of our method in defect localization.

**Figure 20:**
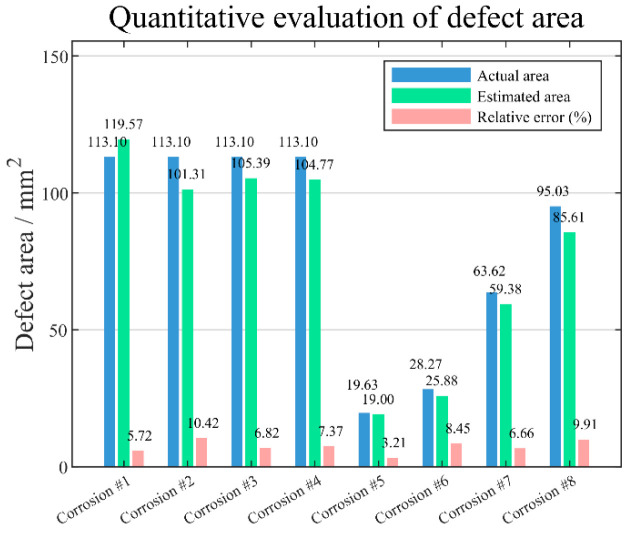
The comparison of the estimated area with the actual area for each defect.

Following the precise localization of the centroid for each defect, the evaluated area can be calculated by the statistical pixels occupied by each defect region after image interpolation. The comparative results between the estimated and actual area of each defect are portrayed in Figure 20, showing that the estimated area is in good agreement with the actual area, with the relative error being less than 10.42%, suggesting that the proposed method provides an accurate and reliable defect area evaluation.

It is noted that the errors in defect localization and area evaluation are mainly attributed to two main factors: (1) specimen processing errors—the rough surface caused by rust removal and sandblasting treatments, as well as the uneven coating of the copolymer adhesive and FBE after melting in the 3LPE structure, leads to variations in coating thickness during specimen preparation; and (2) experimental errors—the vibrations of the probe controlled by the three-axis mechanical scanning stage can lead to lift-off variations, which have a negative impact on experimental data collection.

**Figure 21:**
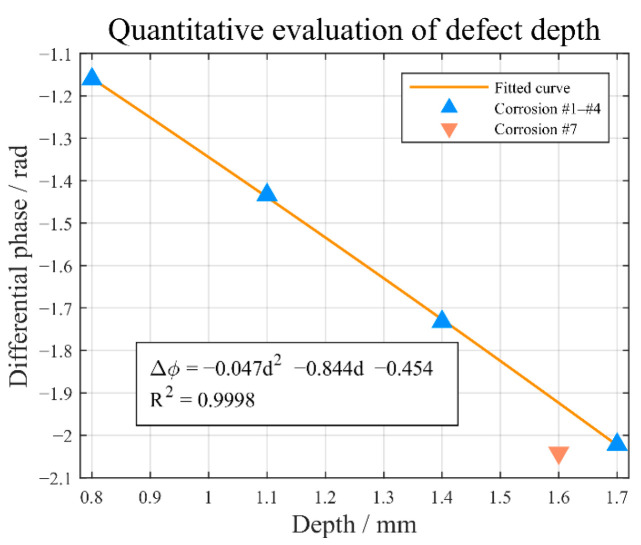
Average differential phase Δ*φ_mean_* against depth *d*.

Since each corrosion region has been accurately identified, the average value of the pixels (representing the differential phase) Δ*φ_mean_* within each corrosion area is computed. The relationship between the Δ*φ_mean_* and the corrosion depth *d* in specimen 1 is illustrated in Figure 21, showing that Δ*φ_mean_* has a distinct monotonic and linear relationship with the artificial FBHs depth *d*. The established fitting function is formulated as follows: $\Delta \varphi_{mean}(d)=-0.047d^{2}-0.844d-0.454$. The average relative error in depth estimation is 0.1%. In parallel, the corrosion depth can be inversely approximated through the function. The estimated depth for defect #7 is 1.71 mm, with a relative error of 6.8%. This is because the variations in defect size and shape induce reflective disturbances that impact the assessment. The above findings indicate that the fitting function above is suitable for depth estimation without a significant loss of evaluation accuracy.

To conclude, the quantitative evaluation results are satisfactory. By integrating near-field microwave frequency-sweeping with phase unwrapping, background subtraction of pipeline and principal component extraction and fusion of image sequences, the high-quality visual imaging and accurate quantitative assessment of external corrosion in 3LPE coated pipelines can be achieved. It is crucial to emphasize that the conclusions presented are based on pipelines with specific coating thickness under controlled laboratory environment. The detection applicability to pipelines with varying diameters and coatings requires additional validation.

## 6. Conclusions and Future Work

This research presents a fundamental feasibility study on the detection, imaging, and quantitative evaluation of the artificial machined FBHs beneath the 3LPE coating of a steel pipeline via phase information obtained from the near-field microwave frequency-sweeping inspection. An improved branch-cut algorithm is proposed for unwrapping the raw phase image sequences, and its superiority over traditional methods is validated concerning unwrapping accuracy and noise robustness. A microwave near-field experimental system is simultaneously established with 3LPE coated pipes containing FBHs to explore hidden corrosion imaging and evaluation. A KDE-based background subtraction method is presented to eliminate the lift-off effect caused by the pipeline surface and further enhance the quality of defect images. The interference of annular artifacts around the defect has been alleviated via PCA-wavelet-based principal components extraction and fusion on a differential phase image sequence, achieving a high-precision evaluation with a relative error below 2.25% for localization and 10.42% for area. The correlation between the depth of corrosion and the average differential phase is ultimately established for performing the depth assessment.

While this study contributes to the microwave detection of pipeline corrosion beneath 3LPE coatings and demonstrates promising basic feasibility, the current approach still exhibits certain technical limitations in practical applicability when compared to the commercial NDT technologies, as detailed below: (1) The idealized artificial defects cannot fully reflect the complexity of natural pitting corrosion, particularly in terms of irregular depth distribution, intricate surface characteristics, and multi-scale morphologies; (2) Although the proposed algorithm offers advantages in accuracy and robustness, its high computational cost and the raster scanning manner using a single probe limit its practicality for rapid screening in large pipeline scenarios; and (3) The dependence on coating properties (e.g., thickness, dielectric constant) and sensitivity to lift-off distance significantly impacts the resolution and sensitivity of microwave near-field detection.

In response to these challenges, future efforts will primarily focus on the practical application and industrial deployment, including but not limited to the following aspects: (1) Optimizing the efficiency of the current unwrapping algorithm by integrating strategies such as Kalman filtering, least squares, and quality-guided techniques, along with parallel computing (e.g., multi-core GPU/CPU cooperation) and hardware acceleration frameworks; (2) Evaluating the detection performance under varying coating thicknesses and typical pipeline operational noise (vibration, temperature gradient, etc.); (3) Developing flexible phased array probes and parallel scanning mechanisms to extend the detection range, along with real-time distance compensation algorithms to reduce lift-off effects; and (4) Exploring the feasibility and application potential of alternative low-power devices, such as oscillators, resonant rings, and microstrip antennas, for real-time monitoring of pipeline natural corrosion under in situ environments.

## Figures and Tables

**Table 1:** Quantitative comparisons under different phase gradient and noise level.

Peaks	*σ*_noise_	Root Mean Square Error (rad)				Signal-to-Noise Ratio (dB)			
		TIE	CPULSI	SRNCP	Ours	CPULSI	CPULSI	SRNCP	Ours
5	0.3	1.425	1.425	1.425	**1.425**	38.842	38.842	38.842	**38.842**
	0.5	1.984	1.984	1.984	**1.983**	35.969	35.970	35.968	**35.971**
	0.8	8.459	4.139	4.414	**3.748**	23.373	31.241	29.023	**32.444**
10	0.3	0.692	0.692	0.692	**0.692**	45.118	45.119	45.119	**45.119**
	0.5	1.323	**1.090**	1.092	1.091	37.076	**41.172**	41.165	41.169
	0.8	5.477	3.241	5.649	**1.654**	27.149	31.707	26.880	**37.547**

**Table 2:** The contribution of principal components.

PCs	Specimen I		Specimen II	
	Component Contribution (%)	Cumulative Contribution (%)	Component Contribution (%)	Cumulative Contribution (%)
PC1	81.37	81.37	70.84	70.84
PC2	9.87	91.24	12.06	82.90
PC3	4.05	95.28	8.10	91.00
PC4	2.15	97.43	3.72	94.72
PC5	1.27	98.71	2.58	97.30

**Table 3:** Evaluation of defect centroid localization (unit: mm).

Defect Number	Actual Location	Evaluated Location	Relative Error (%)
#1	(25.50, 102.00)	(25.19, 102.60)	(1.23, 0.59)
#2	(25.50, 76.00)	(25.50, 76.00)	(0.00, 0.00)
#3	(25.50, 48.00)	(25.48, 48.04)	(0.07, 0.09)
#4	(25.50, 18.00)	(25.62, 17.69)	(0.46, 1.74)
#5	(25.00, 114.00)	(25.00, 114.00)	(0.00, 0.00)
#6	(25.00, 88.00)	(25.00, 88.00)	(0.00, 0.00)
#7	(25.00, 58.00)	(25.31, 57.61)	(1.24, 0.68)
#8	(25.00, 23.00)	(25.04, 22.48)	(0.18, 2.25)

## Data Availability

The data presented in this study are available on request from the corresponding author after obtaining permission of an authorized person.
